# HIV Status Disclosure to Perinatally-Infected Adolescents in Zimbabwe: A Qualitative Study of Adolescent and Healthcare Worker Perspectives

**DOI:** 10.1371/journal.pone.0087322

**Published:** 2014-01-27

**Authors:** Khameer K. Kidia, Zivai Mupambireyi, Lucie Cluver, Chiratidzo E. Ndhlovu, Margaret Borok, Rashida A. Ferrand

**Affiliations:** 1 University of Oxford, Oxford, United Kingdom; 2 University College London, London, United Kingdom; 3 Centre for Sexual Health and HIV/AIDS Research, Harare, Zimbabwe; 4 University of Cape Town, Cape Town, South Africa; 5 University of Zimbabwe College of Health Sciences, Harare, Zimbabwe; 6 London School of Hygiene and Tropical Medicine, London, United Kingdom; 7 Biomedical Research and Training Institute, Harare, Zimbabwe; The University of New South Wales, Australia

## Abstract

**Introduction & Objectives:**

Due to the scale up of antiretroviral therapy, increasing numbers of HIV-infected children are living into adolescence. As these children grow and surpass the immediate threat of death, the issue of informing them of their HIV status arises. This study aimed to understand how perinatally-infected adolescents learn about their HIV-status as well as to examine their preferences for the disclosure process.

**Methods:**

In-depth interviews were conducted with 31 (14 male, 17 female) perinatally-infected adolescents aged 16–20 at an HIV clinic in Harare, Zimbabwe, and focused on adolescents' experiences of disclosure. In addition, 15 (1 male, 14 female) healthcare workers participated in two focus groups that were centred on healthcare workers' practices surrounding disclosure in the clinic. Purposive sampling was used to recruit participants. A coding frame was developed and major themes were extracted using grounded theory methods.

**Results:**

Healthcare workers encouraged caregivers to initiate disclosure in the home environment. However, many adolescents preferred disclosure to take place in the presence of healthcare workers at the clinic because it gave them access to accurate information as well as an environment that made test results seem more credible. Adolescents learned more specific information about living with an HIV-positive status and the meaning of that status from shared experiences among peers at the clinic.

**Conclusions:**

HIV-status disclosure to adolescents is distinct from disclosure to younger children and requires tailored, age-appropriate guidelines. Disclosure to this age group in a healthcare setting may help overcome some of the barriers associated with caregivers disclosing in the home environment and make the HIV status seem more credible to an adolescent. The study also highlights the value of peer support among adolescents, which could help reduce the burden of psychosocial care on caregivers and healthcare workers.

## Introduction

Perinatally-infected adolescents living with HIV/AIDS are a group that presents novel challenges associated with long-term survival with chronic HIV/AIDS [1], including social and cultural factors linked to development and sexual maturation [2]. There is thus a pressing need for tailored, sensitive approaches to providing psychosocial care for HIV-positive adolescents [3], [4].

Due to the scale up of antiretroviral therapy over the last decade, survival of perinatally-infected children has improved dramatically, enabling them to live into adolescence and beyond [5]. As they do so, the issue of informing adolescents about their HIV status arises, a process termed “status disclosure” [6]–[8]. Although status disclosure can also refer to the act of a person living with HIV/AIDS sharing his or her status with others (also known as “public” or “social” disclosure) [9]–[12], in this paper we solely address the issue of telling adolescents about their status and making this status understood. In the literature on pediatric HIV disclosure, a distinction is often made between “full” and “partial” disclosure [6], [8], [13]. In this study, we define full disclosure as having been achieved not only when an adolescent has received all the information about his or her HIV-status but also when he or she entirely understands the ramifications of this information.

Knowing one's HIV status is recognized as an important prerequisite for effective adherence to antiretroviral therapy [14]–[16]. Disclosure has also been associated with a reduction in risk behavior [17]. Furthermore, while there is little evidence that disclosure causes psychological harm and research suggests that it may actually be beneficial for a young person's mental wellbeing [18], a recent study from Zimbabwe showed that learning about their HIV status is still one of the most difficult life events for adolescents living with HIV/AIDS [3]. Despite these facts, the World Health Organization's (WHO) guidelines for HIV status disclosure are limited to children under the age of 12 [8] even though many perinatally-infected children are not disclosed to until they are adolescents [19]. Healthcare workers and caregivers have minimal tailored guidance on how to approach the issue of disclosure to these adolescents except that full disclosure is encouraged and that this should occur in “developmentally appropriate” stages [8]. The WHO recently developed new guidelines for HIV testing and counseling in adolescents [20], however, these guidelines do not address the issue of disclosure *to* adolescents, and only deal with disclosure of adolescents' HIV statuses to others [4]. For disclosure to adolescents, the new guidelines simply defer to the abovementioned existing guidelines for children under 12 [20].

The few data that do exist on disclosure in African settings are focused on younger children [13], [21], or adults [22] and mainly highlights the importance of disclosure for antiretroviral therapy adherence [14]–[16], and the barriers to disclosure [23], [24]. Therefore, to-date, region-specific guidelines on disclosure in Sub-Saharan Africa have often lacked a local evidence base for the best practices described [25], [26] and major international guidelines [7], [8] use evidence from studies in western, developed settings [27]–[30]. Given that the vast majority of perinatally-infected adolescents live in Sub-Saharan Africa [31], and that HIV status disclosure is a complex psychosocial process, it is important to address the issue within specific sociocultural contexts in order to assess the validity of such broad guidelines. In addition, many of the studies described above focus on the perspectives of caregivers [13], [32] rather than those of children or adolescents themselves. An improved understanding of how “full disclosure” is achieved from an adolescent perspective would be vital in the development of future guidelines on HIV counseling tailored for older children and adolescents. In this study we sought to examine healthcare worker and adolescent perceptions of the disclosure process using qualitative interviews and focus group discussions. Given the paucity of knowledge on adolescent-HIV disclosure and the complexity of the process, qualitative methods were ideal for generating hypotheses about the mechanisms involved and for obtaining in-depth perspectives from adolescents dealing with a chronic, stigmatizing illness [33], [34].

## Methods

### Setting

The study was conducted from December 2012 to July 2013 at the HIV clinic of Parirenyatwa Hospital in Harare, Zimbabwe: one of the largest HIV treatment facilities in the country. The center has a specifically tailored adolescent clinic on a weekly basis and an enrolment of 1473 HIV-positive adolescents. Adolescents on treatment who are aware of their status also have access to a clinic-based peer support group.

### Study Design and Sample

In this qualitative study we used a combination of in-depth interviews with perinatally-infected adolescents aged 16–20 (n = 31; mean age = 17) who were aware of their HIV status and two focus group discussions with healthcare workers (n = 15) at the clinic. Adolescents were selected using purposive sampling [35] at their weekly peer support group, which normally has 30–40 adolescents aged 12–20 in attendance. After the study was explained, 3–4 volunteers were chosen per week to obtain a sample of maximum variation [34], [36] across age and gender within the inclusion criteria (16–20 years old and aware of HIV-status). Out of the 31 adolescents who participated, 14 were male and 17 were female. During the interviews, all participants described themselves as being vertically infected.

Healthcare workers for the focus groups were also recruited using maximum variation purposive sampling at their morning staff meeting. These “key informants” [34] included: nurses (with various levels of training), nurse assistants, administrators, and HIV counselors.

### Data Collection

Each adolescent interview lasted approximately 45–60 mins. The English interview guide **(** **Table 1** **)** was translated into Shona (the local vernacular) and back translated to ensure clarity. Adolescents were given the choice of participating in the language in which they felt most comfortable. The two interviewers (KK, ZM) were both Zimbabweans of different gender with experience in social science and health research. The interviewers used the guide to speak to adolescents about their illness learning experiences: how they found out that they were HIV-positive, the significance of this moment, and the knowledge acquisition trajectory that ensued.

**Table 1:** Interview Guide.

1. How has your day been?
2. How did you get into the clinic today?
3. How are you feeling today?
Is there anything with your health that is bothering you?
4. Did you have any idea of your status before the first time that it was explained to you?
How did you obtain this knowledge?
5. Did you ever take pills without knowing what they were for?
6. Why were you tested?
Did you know you were being tested at the time?
7. Think back to the time you were told about your illness Can you tell me what happened.
Who told you that you were HIV-positive?
Where were you told? Describe the setting.
What was your initial reaction?
8. What did you think of the way you were told? Was it the right way? Would you have preferred anything else?
Would you have preferred to have been told sooner? Later?
Would you have preferred to have been told by someone else? (HCWs, family members, combination?)
Would you have preferred to have been told in a different place?
9. Was it important to be told your status?
10. What has changed since then?
11. Do you have friends here at the clinic? Can you tell me about them?
What do they mean to you?
Have you learned anything from them?
Do you go to the support groups?
12. If you were going to tell someone that they were HIV-positive, how would you do it?
13. Is there anything else that you think would be helpful for those that work at this clinic to know about teenagers?
14. Do you have any questions on this topic, or anything else you would like to ask me?

The study used a grounded theory approach for both data collection and analysis [37]. During the data collection period, the two interviewers met before and after each day of interviewing to discuss the emerging themes that surfaced from adolescent narratives. From these discussions, interviewers adjusted future interviews, allowing the interview guide to evolve over the research period and ensuring a constant comparative approach to data collection and preliminary analysis [37]. Interviewers continued to interview participants until no new major themes emerged from the interviews and theoretical saturation had been reached [37].

Each focus group lasted approximately 90 minutes. The focus group moderator (KK) used a modified guide to lead discussions with healthcare workers about the clinical practices associated with disclosure, the role they played in the process, and the challenges that arise with the current system and guidelines.

With permission, interviews and focus groups were audio-recorded and later transcribed verbatim. One interview participant did not consent to audio recording and the interviewer took hand-written notes instead. Additional hand-written notes were taken during focus groups to guide the data analysis process by giving an indication of non-verbal communication and group dynamics during these discussions [38].

### Data Analysis

Again using a constant-comparative approach [37], two members of the research team (KK, LC) with different cultural and professional backgrounds independently reviewed and coded transcripts. Through a line-by-line analysis of transcripts, the two coders assigned labels to contiguous segments of text pertaining to similar ideas (codes). The coders met several times to reconcile emerging codes, allowing them to come up with the first set of 20 codes. A computerized database in MS Excel was then used to aid the data organization and retrieval process.

A research team with experience in medical anthropology, qualitative health research, social work, and adolescent HIV (KK, ZM, LC, RF, CN, MB) met several times in order to organize and discuss emerging themes and concepts until a consensus was reached on the final coding framework. Researchers developed hypotheses on an inductive basis and performed axial coding [39] in order to sort codes into categories and determine how these categories were interconnected. A selective coding process [39] further allowed the research team to piece together concepts from the data in order to develop a descriptive model of the disclosure process for adolescents. The coding tree is provided **(** **Table 2** **)** and key themes of the descriptive model are illustrated using selected quotations in the results section.

**Table 2:** Coding Tree.

Initial Codes	Axial Coding	Selective Coding
How found out	Peer support networks	**Process of disclosure**
Caregivers ill-equipped	Kinship	
Initial reactions		
Suspicions		
Reasons for being tested		
Taking medication without knowing	Medication	**Importance of disclosure**
Adherence		
Poor adherence		
Safe sex	Risk behavior	
Disclosure to partners		
Secrecy	False chronic conditions	**Barriers to disclosure**
Stigma		
Family members (mother, father, other)	Best person	**Preferences**
Healthcare workers		
Early	Best age	
Average		
Late		
Clinic	Best place	
Home		
Public place		
	Desire for the truth	

### Ethical Considerations

Informed written consent was obtained from all adult participants and from the caregivers of participants below the age of 18. Minors below the age of 18 also provided written assent in addition to their caregivers' consent. Ethical approval for this study was obtained from: University of Oxford Interdepartmental Research Ethics Committee, Biomedical Research and Training Institute IRB Board, Joint Research Ethics Committee of the University of Zimbabwe, and the Medical Research Council of Zimbabwe.

## Results

This study uncovered approaches for disclosure to HIV-positive adolescents that were highly varied and that did not follow any standard protocol. We found that although healthcare workers encouraged parents to initiate disclosure to their children in the home environment, adolescents themselves preferred a clinical setting. In the clinic, adolescents had access to accurate information from healthcare workers and an environment that made the illness seem more real. Furthermore, many adolescents did not learn much when they were first told about their illness. Instead, they turned to support from peers within the clinic, among whom they felt comfortable to share experiences and learn about HIV/AIDS.

### Healthcare workers encourage home-based disclosure

Healthcare workers encouraged caregivers to disclose to their children in the home environment and prioritized this method over in-clinic disclosure: *“The first preference is given to the parents to do it bit by bit at home. If they fail, counselors are there to disclose”* (counselor A, FGD1).

Healthcare workers explained that they did not have enough time to spend with each child, that this was “their system,” and that it was “written in [their] manuals.” They also assumed that adolescents were old enough to understand HIV-related concepts and that they had been explained these concepts when they were younger, during post-test counseling. Therefore, rather than being involved in the first communications with adolescents about their illness, healthcare workers tended to play a delayed and auxiliary role in the adolescent disclosure process whereby they reinforced or corrected information that was communicated by caregivers at home:

*“Next week, when they come for a visit, we'll ask them, ‘Did you disclose to the child?’ If they say ‘Yes,’ we'll start a session again to reinforce to the child what HIV is, what it means. This is an individual session after the parents have disclosed—to see if the child understands what HIV is, to see if he or she is not stressed and if they accept it” (counselor B, FGD2).*

It was clear from information provided by adolescents that encouraging disclosure in the home environment could lead to suboptimal outcomes: “*My grandmother told me at home. I was watching TV. My grandmother came up to me and said, ‘Hey, A, do you know that you're HIV-positive?’ I said, ‘Okay.’ She said it twice: ‘You're HIV-positive.’ I just said, ‘Okay’”* (boy A, 18).

### Caregivers conceal the truth despite adolescents' abilities to understand

Although we were unable to interview caregivers in this study, we indirectly learned about their beliefs from the healthcare worker focus groups. Even though healthcare workers encouraged parents to disclose at home, they identified several issues with getting parents to do so. One of the key challenges was that many caregivers believe that their child was too young to grasp the concepts associated with HIV/AIDS:

*Some parents feel as though they are not ready to disclose a status to a child because they feel maybe he or she is too young and can't take it. Maybe it will discourage the child. They have a lot of mixed feelings concerning disclosing a status to their children, so they will tell you they are not ready to disclose (counselor C, FGD 2).*

Second, caregivers worried that children were not mature enough to keep a secret, and might share it with others in the community: “*They fear that if the child is disclosed to, he will go about in the streets or at school telling others and other relatives that don't know that the parents are positive. So they will be stigmatized or discriminated against as a family, so they fear telling their children” (counselor D, FGD 2).*

Hence, when tasked with disclosing to their children in the home environment, many parents evaded the issue by lying or not revealing the full truth about the illness. Despite this, many adolescents uncovered their status by making inferences from their surrounding or situation: *“My mother was lying to me saying I have a heart problem, I have a hole in my heart. So I decided to say, ‘Okay.’ But I knew. I knew that when I was coming here I was HIV-positive” (boy B, 17).*

### Adolescents want to be told the truth and prefer disclosure in a healthcare setting

While many caregivers concealed HIV-statuses from adolescents, HIV-positive adolescents expressed a strong desire for open and truthful communication: “*I think you should just be frank with [adolescents]* … *Don't make the child suspect or have second thoughts*… *if it turns out to be positive*… *just tell them you're HIV-positive and explain that being HIV-positive is not the end of the world” (girl A, 17)*.

In addition to wanting to be told the truth about their illness, many adolescents expressed clear preferences about how this should occur. They described the ideal scenario for being told about their HIV status as a healthcare setting that included communication with a doctor, nurse, or counselor. For adolescents, both the clinical space and the presence of a healthcare worker served to reinforce the reality of the illness: “*I wanted to be told at the clinic just so I know that it's really true, that I've been tested, and it's true” (girl B,17)*. Adolescents said that having a healthcare worker present was also important for obtaining accurate information about HIV/AIDS and answers to the many questions they might have at that time of uncertainty: *“I wanted to be told by a nurse or a doctor because they are the ones who would be able to explain all about your disease, like what HIV is” (boy C, 18)*.

### Adolescents struggle to understand at first and learn more from peers

Although most adolescents were able to vividly recall the event when they were first told that they were HIV-positive, many said that they did not fully understand what HIV/AIDS was at the time, or that they were too shocked by the news to grasp any of the HIV/AIDS-related information that was being conveyed: “*They kept on talking until eventually they told me. But when they told me I did not know what positive or negative meant at the time… I didn't even care… On the day I did not understand anything, I did not feel anything” (boy D, 18–disclosed to at age 16).*

Instead, adolescents learned a great deal about HIV/AIDS and its ramifications from their more-experienced peers during peer support groups and sharing sessions: *“I learnt a lot from a support group I went to at the state medical hospital. One of the counselors at the clinic took me there and I learnt much there” (girl C, 19—disclosed to at 18 years).*

Adolescents also explained that once they attended support groups and became more knowledgeable about HIV/AIDS, they could go on to teach others about the importance of adherence to medications, avoiding risk behavior, and staying healthy:

*I did not believe because when I was first tested I came on a day when there were only adults and I did not see any other children that day… I was thinking of committing suicide…at the time I did not know much. Also I had not met with other adolescents and I was alone. That is when I started going and attending support groups. Now I am teaching others and telling them that they are HIV-positive. I tell them what to do, for example take your pills at 6. You must take them exactly on time and I tell them the food they must eat (boy E, 16).*

Healthcare workers confirmed the helpful role of adolescents themselves in the disclosure process and noted that HIV-positive adolescents formed a highly cohesive community centered on support groups and informal social relations at the clinic. When healthcare workers were having difficulties disclosing to children who were struggling to grasp all the concepts associated with being HIV-positive, more experienced adolescents would be recruited to assist in the process by providing support and explanations in age-appropriate terminology:

*We make the other adolescents who are aware of their status disclose to the younger ones… we make the other adolescents who are mature encourage the fellow adolescent to adhere well and to explain. If it is explained by one who is in the same situation, they understand better (counselor B, FGD1).*

## Discussion

To our knowledge, this is the first study that has explored the preferences of adolescents in the HIV disclosure process and the first to highlight the roles of peer support and healthcare settings in assisting the ongoing process of disclosure for this age group.

Although adolescents expressed a preference for being disclosed to in the clinic, healthcare workers have been guided to encourage parents to disclose in the home environment. This method created barriers to the initiation and completion of this process and healthcare workers described having to reinforce or correct poorly conveyed concepts from caregivers who attempted to disclose in the home environment, but failed to do so adequately. While healthcare workers attributed these practices to manuals on testing and counseling, we found no such indication in local guidelines [25], suggesting that healthcare workers may require additional support in using current guidelines and/or that current guidelines are insufficient.

Even though adolescents expressed a desire to know the truth about their HIV-status, caregivers often concealed statuses or misinformed adolescents, which led to adolescents discovering their HIV status accidentally. This resulted in a knowledge gap for adolescents who miss the opportunity to learn important concepts in a standardized fashion that would ensure they have understood all the ramifications of their HIV-status. Apart from the WHO guidelines for disclosure to children under 12 years [8], there are currently minimal guidelines on how best to inform these adolescents of their status and make it understood.

We discovered that one way that adolescents filled this knowledge gap was by using information gathered from their HIV-positive peers. More experienced adolescents are capable of using age-appropriate terminology to explain important HIV/AIDS concepts to those that have recently been told about their HIV-status. Practices described by both healthcare workers and adolescents underscored the importance of peer support among HIV-positive youth in the disclosure process.

Conveying comprehensive understanding of complex concepts requires both repetition and reinforcement [40]. It is therefore unsurprising that HIV-positive youth learn about HIV/AIDS-related concepts from their peers in addition to official communications from when they are first told about their illness. However, the lack of formal recognition of the utility of peer support specifically for disclosure to adolescents living with HIV/AIDS has led to the development of guidelines and practices that are centered on the first event of telling a young child that he or she is HIV-positive, rather than the important learning trajectory that ensues among older children and teenagers. The idea that disclosure is not a single event, but rather an “ongoing process” is not a new one [6]–[8], [25], [41]. However, based on the data gathered in this study, we suggest that through shared experiences that reinforce HIV-related information, peer support among adolescents assisted the ongoing process of disclosure once it had been initiated. Rather than perceiving disclosure simply as an “ongoing process” consisting of a linear series of events involving one adolescent, we argue that the process was a dynamic one that encompassed an entire peer group. As adolescents learned more about what it meant to be HIV-positive, they became more confident in guiding their peers. Since our study highlights the unique capacity of adolescents to assist each other in the disclosure process, it describes disclosure to adolescents as a process that is distinct from disclosure to younger children and therefore one that requires more age-specific guidelines than those currently offered by the WHO [8].

The findings from our study are limited in several ways. First, our adolescent participants were selected from and interviewed at an urban clinic in the capital city. It is likely, therefore, that the perspectives of our interviewees are biased towards an interpretation of disclosure as a process that is best conducted in a clinical setting, where participants had access to an organized peer support group. A study that incorporated interviews from marginalized, rural communities and non-clinical settings would have provided a sample with a greater variety of perspectives. Nonetheless, our study did not seek to describe the viewpoints of a representative sample, but rather generate some hypotheses about how perinatally-infected adolescents come to understand their positive HIV-status.

Second, in addressing disclosure in the home environment, due to time and funding limitations, we did not have the opportunity to interview caregivers and instead obtained their perspectives via those of healthcare workers. Since some studies that reflect the perspectives of caregivers in the disclosure process have been conducted before [13], [32], we chose to focus on the perspectives of adolescents as no previous studies have done so. Further studies that incorporate perspectives from caregivers who have disclosed, as well as those who have not yet done so, would still be useful and we recommend this as an important area for future research.

For these reasons and similar issues related to our small, isolated sample, the generalizability of our findings is greatly limited. Further studies with larger, more varied samples that include caregiver perspectives as well as those of perinatally-infected youth in rural and peri-urban settings are vital for a holistic representation of adolescent status disclosure. Once a wide range of hypotheses regarding adolescent preferences for status disclosure has been developed, quantitative surveys could help confirm this data for use in the development of more specific disclosure guidelines for this age group.

In light of the emerging adolescent HIV epidemic, the development of tailored guidelines for disclosure to HIV-positive adolescents should be prioritized. In this endeavor, an appreciation of the setting in which disclosure takes place as well as the ability of older children to support each other should be considered. The dynamic nature of HIV disclosure to adolescents that we describe in this study suggests that peer-mentoring systems, which incorporate the role of experienced adolescents into the difficult process of conveying HIV/AIDS-related concepts to young people, could be useful for delivering information in age-appropriate terminology and idioms. Such systems may also help reduce the burden on healthcare workers and caregivers involved in the psychosocial support of HIV-positive youth.
